# Encountering the unicorn – abscopal effect after fractionated stereotactic radiosurgery for brain metastasis in renal cell carcinoma: a case report and review of the literature

**DOI:** 10.3332/ecancer.2025.1964

**Published:** 2025-08-12

**Authors:** Nishana Abbas, Durgapoorna Menon

**Affiliations:** 1 Department of Radiation Oncology, Tata Memorial Hospital, Mumbai 400012, India; 2 Department of Radiation Oncology, Aster Medcity, Cheranellore, Kochi 682034, India

**Keywords:** clear cell renal cell carcinoma, abscopal effect, fractionated stereotactic radiosurgery, brain metastasis, case report

## Abstract

Clear cell renal cell carcinoma (ccRCC) is a malignancy with a diverse clinical presentation, often characterised by its resistance to conventional therapies. We present the case of a 53-year-old Indian male diagnosed with ccRCC, pT3aN0M1exhibiting lymphovascular invasion and non-contiguous tumour deposits in the left adrenal gland. Following an initial course of Pazopanib and subsequent Everolimus, the disease progressed. Notably, just 3 months after receiving fractionated stereotactic radiosurgery (FSR) for a cerebellar metastasis, spontaneous regression was observed at a distant skeletal site. This case highlights the significance of considering FSR as a therapeutic option for selected patients with ccRCC, as well as the potential role of an abscopal effect to impact the course of this aggressive malignancy.

## Background

Clear cell renal cell carcinoma (ccRCC) is the most common subtype of renal cell carcinoma, known for its potential for aggressive growth and metastatic dissemination [1]. Since many decades, the concept of the ‘abscopal effect’ has garnered considerable attention in the context of cancer treatment. This phenomenon has been described when localised radiation therapy triggers a systemic immune response, causing distant metastatic lesions to regress, even if they were not directly targeted by the radiation [2–5].

Here, we present a rare case of ccRCC who underwent fractionated stereotactic radiosurgery (FSR) for a metastatic lesion in the cerebellum. Remarkably, imaging after just 3 months demonstrated a probable abscopal effect, with spontaneous regression of the previously detected distal skeletal lesion.

## Case description

The patient, a 53-year-old Indian male, was initially evaluated for a left renal mass and underwent a Laparoscopic Left Nephrectomy. Pathologic evaluation confirmed a ccRCC. The histopathology report revealed lymphovascular invasion and non-contiguous tumour deposits in the left adrenal gland, Stage pT3aN0M1. A postoperative PET CT scan showed no FDG-avid residual disease in the surgical bed or other areas. But there were CT-detected nodules and thickening of fascia in the surgical bed, which suggested a cautious outlook. Since ccRCC is typically FDG non-avid, the patient was initiated on Pazopanib, an oral tyrosine kinase inhibitor.

After 6 months of Pazopanib therapy, a follow-up PET CT scan revealed disease progression. There were multiple FDG-avid soft tissue deposits, including in the left side of the diaphragm, the spleen, the anterior abdominal wall, the iliopsoas, a para-aortic lymph nodal mass and a metabolically active FDG-avid sclerotic lesion in the second lumbar (L2) vertebra. In view of this, the patient was switched to Everolimus, an mTOR inhibitor.

**Figure 1.**
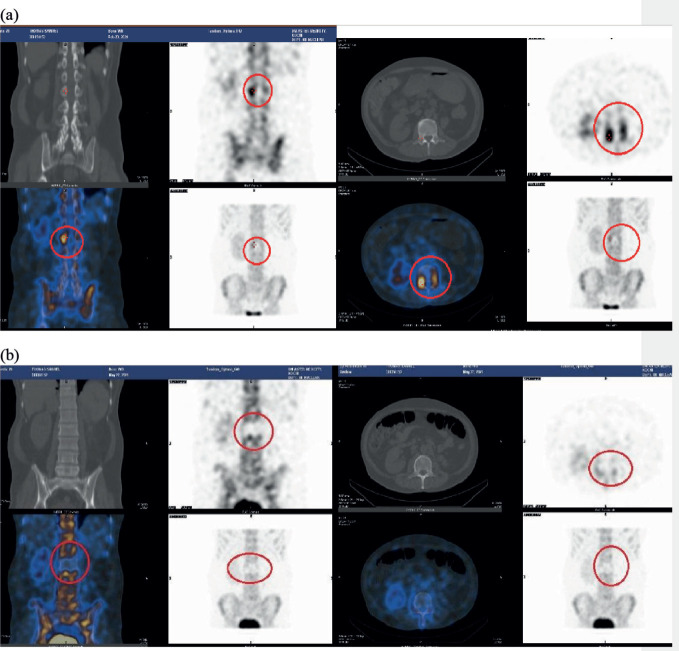
(a): SPECT images in metastatic RCC showing increased tracer uptake over the L2 pedicle on the right. (b): SPECT images 3 months after intracranial FSR, showing no tracer uptake over the L2 pedicle on the right.

However, 6 months after starting this therapy, he presented with symptoms of nausea, vomiting and mild lower back pain. Magnetic resonance imaging of the brain revealed a solitary 2.5 cm lesion in the right cerebellum, suggestive of brain metastasis. Additionally, a bone scan showed increased tracer uptake in the right pedicle of the L2 vertebrae, (Figure 1a).

**Figure 2.**
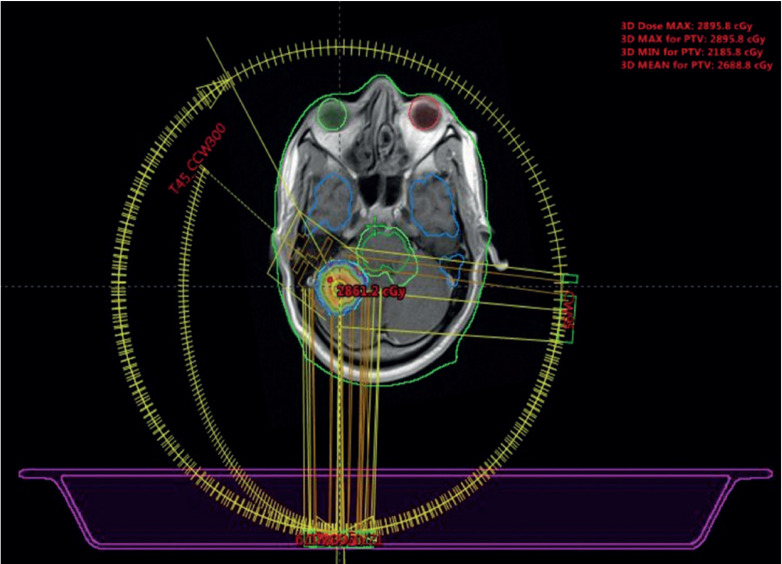
Intracranial FSR treatment field for cerebellar metastasis.

Since the right kidney was the only functioning kidney, and in view of the relatively small size of the spinal lesion which was only mildly symptomatic, the multidisciplinary team (MDT) decision was to withhold radiation therapy (RT) to the spine. The patient underwent FSR to the right cerebellar metastasis, receiving a total dose of 27.5 Gy administered in five daily fractions, each delivering 5.5Gy (Figure 2). All systemic therapy was stopped and it was decided to reassess with imaging at 3 months. As per our institutional protocol, a PET scan is ordered only at 6 months post treatment, especially in the palliative setting. Hence, 3 months after FSR, a reassessment bone scan was done that demonstrated a complete response in the non-irradiated site, the L2 vertebral pedicle (Figure 1b). This dramatic response was deemed consistent with the abscopal effect.

## Discussion

Clear cell renal cell carcinoma is a subtype of renal cell carcinoma known for its resistance to conventional therapies and propensity for metastasis. This case report provides a rare insight into the treatment journey of a ccRCC patient who experienced an abscopal effect at just 3 months after FSR for a brain metastasis. The abscopal effect is a phenomenon where localised radiation therapy to one tumour site induces an immune response that leads to the regression of distant, non-irradiated tumour sites.

Radiation therapy, by causing tumour cell death and the release of tumour neoantigens, can stimulate the immune system [4]. This causes activation of T cell responses that recognise and attack cancer cells leading to the Abscopal Effect. Fractionated regimens activate immune pathways like cGAS–STING, which leads to type I interferon (IFN-1) production, dendritic cell activation and T-cell priming whereas higher single doses (e.g., >20 Gy) may induce TREX1, which degrades cytosolic DNA, suppressing immune activation. Thus, the dose per fraction may be more critical than the total dose in determining immune effects. However, in clinical trials, both fractionation regimens have been shown to induce systemic antitumour response when combined with immunotherapy agents like anti-CTLA-4 or anti-PD-1/PDL-1 agents by overcoming immune checkpoints. RT may also induce immune sensitisation through upregulation of FAS, ICAM1, MHC-I and chemokines like CXCL9/10/16 – a phenomenon akin to a 'vaccine-like effect' [5]. It is still difficult to determine which patient will exhibit an abscopal effect. Hence predictive biomarkers that influence immunogenicity like absolute lymphocyte count, wild-type p53 expression, mutant KPNA2 downregulation and γH2AX may help to identify patients who might exhibit abscopal effect in patients receiving radiotherapy. Additionally, the Vigil vaccine, an autologous GM-CSF–secreting tumour vaccine with TGF-β knockdown, shows promise in priming anti-tumour immunity and enhancing abscopal potential [6]. Although the abscopal effect triggers both our imagination and immunity alike, it was always considered a unicorn by practising oncologists. However, the phenomenon has garnered significant interest in recent years with a windfall of case reports that have likely resulted from the widespread use of immunotherapy combined with the high doses of radiation delivered using stereotactic techniques [5].

In our patient, the ccRCC was initially FDG non-avid, making it challenging to monitor disease progression and response to therapy using conventional imaging techniques. The use of targeted therapies such as Pazopanib and Everolimus demonstrated limited success, underscoring the need for alternative treatment approaches. FSR (Radiotherapy) resulted in a probable abscopal effect in a very short while as evidenced by the complete response observed at the non-irradiated L2 vertebra. This finding raises important questions about the role of immunity in fighting ccRCC and the potential therapeutic benefit of harnessing the abscopal effect. Additionally, although such occurrences are rarely reported, the switch over to Everolimus prior to the FSR may have played a role in enhancing the abscopal effect, as mTOR inhibitors have been implicated in modulating immune responses [7].

Although SPECT-CT has lower sensitivity in RCC than PET-CT, the absence of CT abnormalities both at baseline and follow-up suggests that this may have been a marrow-based lesion [8]. ccRCC is frequently resistant to treatment, and the prognosis for advanced-stage disease is often poor. The clinical significance of the abscopal effect in metastatic ccRCC is unknown. However, the incidence of abscopal responses in renal cell carcinoma patients remains low, at around 0.3% [9]. Table 1 highlights well-documented cases of the abscopal effect in metastatic RCC following both conventional and ablative radiation therapy. These cases demonstrate the potential for favourable outcomes following tumour regression. Notably, none of these patients received systemic treatment at the time of radiation therapy or adjuvant treatment afterward. Interestingly, most reported cases on abscopal effect were on renal cell carcinoma, Non-Small Lung Cancer (NSCLC), melanoma and lymphoma-traditionally regarded as immunogenic. Nevertheless, the abscopal effect has been observed across a range of tumour types, suggesting the potential for the broader application of radiation therapy as an immunomodulatory tool. Its frequency, durability and therapeutic impact are now being more formally explored through clinical trials.

Table 2 highlights case reports and series of abscopal effect in other sites following radiation therapy. In a patient-level meta-analysis of individuals exhibiting the abscopal effect, the 5-year overall survival and 5-year progression-free survival were reported to be 63% and 45% respectively. On univariate analysis none of the variables were statistically significant to predict duration of response or overall survival [34].

In a series of metastatic breast cancer patients treated with stereotactic body radiotherapy (SBRT) without concurrent immunotherapy, approximately 25% exhibited the abscopal effect. These patients with the abscopal effect experienced a positive impact on overall treatment outcomes, with a promising 1-year progression-free survival rate of 70% [35]. Additionally, a phase II prospective study in NSCLC demonstrated the efficacy of RT in overcoming acquired immunotherapy resistance when administered concurrently with immune checkpoint inhibitors with acceptable toxicity [36]. Notably, in these studies, regardless of the RT dose or volume, the addition of hypofractionated RT in the metastatic setting demonstrated an immune-boosting effect.

The potential for SRS-induced abscopal effects could open new avenues for treatment and significantly impact patient outcomes. Furthermore, the successful management of brain metastasis in a ccRCC patient through FSR underscores the importance of individualised treatment plans, especially in cases where patients have limited treatment options due to their medical history and the nature of their disease. In this case, the MDT made a thoughtful decision to opt for FSR to treat the brain metastasis and to observe the spinal lesion, ultimately resulting in a positive clinical outcome.

It is important to acknowledge the limitations in analysing the abscopal effect, due to reporting bias and the concurrent use of other treatments. Despite the promise of the abscopal effect, its occurrence remains infrequent and predicting which patients will experience it remains a challenge. Additionally, the optimal sequencing and dosing of SBRT and immunotherapy require further investigation. As the understanding of the effect continues to evolve, further research and clinical trials may shed light on its potential application in the management of ccRCC and other malignancies.

## Conclusion

Clear cell renal cell carcinoma remains a challenging malignancy to treat, especially in advanced stages. We report a rare ccRCC patient who achieved complete response at a non-irradiated skeletal site at just 3 months post radiation, raising important questions about the immune-mediated responses in ccRCC and the potential role of the abscopal effect in its management. The abscopal effect in itself is a fascinating concept – combining immunotherapy with the high doses of radiation delivered using stereotactic technology has demonstrated considerable promise in triggering this phenomenon. As research in this field advances, a deeper understanding of the abscopal effect and the factors influencing it will likely lead to improved patient outcomes and a brighter future for those with metastatic Renal Carcinomas.

## Conflicts of interest

None.

## Funding

The authors have nothing to disclose.

## Author contributions

Conception and design: Durgapoorna Menon.

Data collection: Nishana Abbas.

Data analysis and interpretation: Nishana Abbas.

Manuscript writing: Nishana Abbas.

Final approval of manuscript: Durgapoorna Menon.

## Figures and Tables

**Table 1.** Case reports of abscopal effect in metastatic RCC.

Author	Age [years]	Year presented	Gender	Primary treatment	RT site	RT-dose/fraction	BED	Areas of abscopal regression	Time interval	Duration of response	Patient outcome
Fairlamb [10]	73	1981	F	Nephrectomy	Kidney	40 Gy/15 fx	51.4	Lung metastases	<12 months	39 months	Alive without disease
MacManus *et al* [11]	58	1994	M	Radiation	Kidney	20 Gy/10 fx	24	Lung metastases, mediastinal nodes	6 months	11 months	Death related to disease
Wersäll *et al* [12]	83 64 69	2009	F F M	Radiation Nephrectomy Nephrectomy	Kidney Lung lesion Lung lesion	32 Gy/4 fx - 30 Gy/2 fx	57.6 - 75	Lung metastases Lung metastases Lung metastases	- 6 months 4 months	24 months 54 months 24 months	Alive with stable primary Alive without disease Alive without disease
Ishiyama *et al* [13]	61	2012	M	Nephrectomy	Brain and spine	Brain 18 Gy single Spine 40 Gy/5 fx	50.4	Lung metastases	4 months	34 months	Alive
Van de Walle *et al* [14]	66	2016	F	Nephrectomy	Neck	39 Gy/13 fx	50.7	Lung metastases, skin nodule	4 months	17 months	Alive with liver metastases – on mT OR
Our case report	53	2024	M	Nephrectomy	Brain	27.5 Gy/5 fx	85.7	Spine lesion	3 months		

Abbreviation’s: BED, Biologically effective dose; assuming an α/β ratio of 10, fx, fraction

**Table 2.** Case reports of abscopal effect in other cancers sites after radiotherapy.

Author	Age [years]	Year presented	Gender	Primary treatment	RT site	RT- dose/ fraction	BED	Areas of abscopal regression	Time interval (months)	Duration of response (months)	Patient outcome
**NSCL**C											
Golden *et al* [15]	64	2013	M	CT f/b RT f/b IT	Liver mets	30 Gy/5 fx	48	Other liver mets, bone	6	12	Alive without disease
Siva *et al* [16]	78	2013	F	CTRT	Lung mets	26 Gy/1 fx	93.6	Adrenal, bone	12	15	Alive with disease
Bitran [17]	62	2019	F	CT f/b RT f/b IT	Lung primary	27 Gy/9 fx	35.1	Adrenal	5	54	Alive without disease
Lin *et al* [18]	71	2019	M	CT f/b RT f/b IT	Right parietal lobe	48Gy/8 fx	76.8	Lung	6	19	Alive with disease
Britschgi *et al* [19]	47	2018	M	RT+IT	Lymphnode	18 Gy/3 fx	28.8	Lymphnode	2.5	24	Alive without disease
Katayama *et al* [20]	63	2017	M	RT alone	Brain & Bone mets	45 Gy/15 fx, 30 Gy/10 fx	58.5	Lung	1.5	9	Alive with disease
Hamilton *et al* [23]	47	2018	M	RT+Sx	Brain mets	25 Gy/5 fx	37.5	Lung, lymphnode	1	7	Alive without disease
Cong *et al* [24]	64	2017	F	RT+IT	Lungs	37.5 Gy/5 fx	65.6	distant lung mets	10	-	Alive without disease
**Melanoma**											
Postow *et al* [25]	33	2012	F	RT+IT	Paraspinal mass	28.5 Gy/3 fr	55.5	Spleen, lymphnode	4	10	Alive without disease
Stamell *et al* [26]	67	2012	M	RT alone	cutanous	24 Gy/3 fr	43.2	distant skin mets	8	36	Alive without disease
Sullivan *et al* [27]	68	2013	M	RT+IT	Brain mets	18 Gy/1 fr	50.4	Bone, lymphnodes	12	18	Alive without disease
Hiniker *et al* [28], Gutkin *et al* [29]	-	2012, 2018	-	RT+IT	Liver mets	54 Gy/3 fr	151	Left axillary LNs	5	6.5 years	Alive without disease
**Lymphoma**											
Lakshmanagowda *et al* [30]	65	2009	F	CT f/b RT	Axillary LN	24 Gy/12 fr	28.8	Neck node	2 weeks	6	Alive without disease
Robins *et al* [31]	59	1981	F	CT f/b RT	Right kidney	20 Gy/10 Fr	24	Left Kidney	2 weeks	4	Death -disease progression
Hidaka *et al* [32]	88	2017	F	RT alone	Left buttock, left upper arm	32 Gy/8 fr, 24 Gy/6 fr,	44.3,33.6	Anterior chest, thigh and lower leg skin lesions	1	6	Death -disease progression
Antoniades *et al* [33]	44	1977	M	RT alone	Mediastinum, lung, neck, Waldeyer’s ring LNs.	30 Gy/20 fr	34.5	Para aortic & pelvis LNs	-	-	Alive without disease

Abbreviations: BED, Biologically effective dose; assuming an α/β ration of 10, fr, fraction; RT, Radiotherapy; CT, Chemotherapy; IT, immunotherapy; Sx, Surgery; Mets, metastasis; LNs, Lymph nodes
